# Phosphate-solubilizing and polymerizing bacteria enhance phosphorus availability and growth of rice

**DOI:** 10.3389/fmicb.2025.1700135

**Published:** 2025-12-08

**Authors:** Meiying Yang, Xueting Qin, Chengcai Yue, Qi Wu, Ping Tian, Zhihai Wu, Xiaoshuang Wei, Lei Wu

**Affiliations:** 1 College of Life Sciences, Jilin Agricultural University, Changchun, China; 2 Faculty of Agronomy, Jilin Agricultural University, Changchun, China; 3 National Crop Variety Approval and Characterization Station, Jilin Agricultural University, Changchun, China

**Keywords:** rice, transcriptome, plant growth, bacterial phosphate solubilization, phosphoste accumulating bacteria

## Abstract

**Introduction:**

Phosphorus (P) is a limiting nutrient in soil-plant nutrient cycling, functional microorganisms can promote P transformation. While previous studies have mainly focused on either phosphorus solubilization or phosphorus aggregation by bacterial strains, few address both aspects simultaneously.

**Methods:**

In this study, we screened Acinetobacter johnsonii (NHP4a-2) and Pseudomonas glycinae (NHP4b-2) strains, both of which can effectively solubilize and polymerize P. Their performance was evaluated by measuring phosphate solubilization capacity, polyphosphate accumulation levels, and the activity of related enzymes (polyphosphate kinase, extrapolyphosphatase, and glucose dehydrogenase). A high-phosphorus/low-phosphorus alternating culture system was employed to simulate aerobic-anaerobic cycles, monitoring phosphorus uptake and release dynamics. Transcriptomic analysis revealed the molecular mechanisms underlying their phosphorus solubilization, and their growth-promoting effects were validated through rice pot experiments.

**Results:**

The phosphorus solubilization capacity of NHP4a-2 is 33% that of NHP4b-2. Under alternating high- and low-phosphorus conditions, the aerobic phosphorus uptake and anaerobic phosphorus release functions of the two bacterial strains. In addition, the polyphosphate kinase, Exopolyphosphatase, and Glucose dehydrogenase enzyme activities of strain NHP4b-2 were higher than those of strain NHP4a-2. Transcriptome analyses show that NHP4b-2 exhibited significant upregulation of 93 differentially expressed genes and downregulation of 264 differentially expressed genes under phosphate solubilization conditions. ATP metabolism, oxidative phosphorylation, and glycolysis/gluconeogenesis pathways supply ATP and acidic compounds to the strain, thereby supporting its phosphate solubilization function. NHP4b-2 exhibits stronger polyphosphate accumulation capabilities than NHP4a-2. This strain absorbs and stores increasing amounts of phosphorus as phosphorus concentration rises. Under low-phosphorus conditions, it releases phosphorus to support plant growth. Compared with the NHP4a-2 treatment group, the NHP4b-2 treatment group exhibited increases of 6.3, 26.3, 13.3, 25.4, and 56.9% in rice seedling plant height, root length, fresh leaf weight, fresh root weight, and phosphorus content, respectively, positively impacting the growth of the rice seedlings.

**Discussion:**

The results of this study indicate that strains possessing both high-efficiency phosphorus solubilization and phosphorus accumulation capabilities achieve effective phosphorus activation and storage by regulating key metabolic pathways, significantly promoting plant phosphorus uptake and growth. This study provides valuable insights into sustainable phosphorus management in agriculture and may have future applications in various agricultural settings.

## 1 Introduction

Phosphorus (P) is a vital biogenic element essential for the biosynthesis of various compounds in plants (Pang et al., 2024; Zhang et al., 2025), it is positively influencing crop growth and development (Bai et al., 2020). Insufficient P nutrition in crops hinders the biosynthesis of these processes, ultimately reducing the photosynthetic rate (Kafle et al., 2019), which greatly limits the development and productivity of plants (Chen et al., 2021). Phosphorus also plays a crucial role in enhancing root branching and strength (Cheng et al., 2023; Hu et al., 2024). In addition, phosphorus aids in seed germination, early ripening of crops like grains and legumes (Zeng et al., 2022), and nitrogen fixation. Moreover, it plays a significant role in maintaining high crop yields and desirable crop characteristics (Benmrid et al., 2014). The speciation of P in soils determines its availability and mobility (Li H. P. et al., 2023; Li Z. et al., 2023; Torres et al., 2024). Inorganic phosphate (Pi) is the only form that plant roots can assimilate (Wei et al., 2023). In addition, soil contains a proportion of organic P, but most of it is ineffective and inaccessible to crops. This limited availability of P compounds makes P a restricting nutrient in the soil–plant nutrient cycle (Percival et al., 2020). Heavy fertilization of the soil is a common approach to addressing this issue. However, exogenous P is often associated with low utilization efficiency, leading to wasted P resources and the accumulation of legacy P in the soil (Cordell et al., 2009; Xiong et al., 2017; Yu et al., 2021). Nutrient leaching from the soil can cause eutrophication in aquatic ecosystems (Huang et al., 2017; Pereira et al., 2018). In addition, excessive use of P fertilizers can deplete the soils’ capacity to retain P, increasing the risk of P loss (Yan et al., 2017; Li et al., 2021). Therefore, finding ways to enhance soil P availability and activate recalcitrant P in soils can help to reduce the demand for phosphate rocks (Li et al., 2016). Biological methods that use microorganisms as P activators have been suggested as potentially the most eco-friendly and cost-efficient approaches to achieving this goal compared to other strategies (Zhu et al., 2018; Li H. P. et al., 2023; Li Z. et al., 2023; Zhang et al., 2025).

Microorganisms play a crucial role in the transformation between organic and inorganic P in soils (Joan et al., 2017). Among these microbes, two types of functional bacteria involved in soil cycling are phosphorus-solubilizing bacteria (PSB) and phosphorus-accumulating bacteria (PAB) (Li H. P. et al., 2023; Li Z. et al., 2023). PSB play a multifunctional role in enhancing soil P utilization efficiency, promoting crop growth and development, and aiding in stress resistance (Fan et al., 2017). They make P available through the solubilization of insoluble mineral phosphates and/or the mineralization of organic P (Delgado et al., 2014; Taurian et al., 2009; Liu et al., 2023). Extensive efforts have been made to isolate PSB for agricultural applications (Devi et al., 2022; Fatima et al., 2021). Biofertilizers containing halotolerant PSB have been proposed as an eco-friendly alternative to chemical P fertilizers, supplementing available P for crop production in saline areas (Dey et al., 2021). Khourchi et al. (2023) investigate the crucial role of PSB in enhancing P acquisition and phosphorus use efficiency (PUE) of wheat plants. Similarly, Massucato et al. (2022) aimed to select and validate bacterial strains that solubilize phosphorus and promote maize growth. The effective application of PSB can release the accumulated P left by traditional P fertilizer in soils, thereby avoiding environmental damage that results in soil hardening and water eutrophication (Billah et al., 2019; Etesami, 2020). Multiple P removal metabolic pathways have been identified. For instance, some bacteria can effectively absorb phosphate and store it as intracellular polyphosphate (polyP) under aerobic conditions (Saia et al., 2021; Zhao et al., 2022). Meanwhile, other bacteria accumulate phosphorus in extracellular polymeric substances (EPS) through assimilation or by inducing phosphate precipitation (Jiang et al., 2018). Currently, research on PAB has mainly focused on enhanced biological phosphorus removal in wastewater treatment, with limited attention given to their agricultural applications (Li H. P. et al., 2023; Li Z. et al., 2023). Despite this limited research, PAB characteristics could potentially be used to reduce P leaching losses in agriculture. However, further experimentation is needed to validate this potential.

Microbial biomass P is the most labile component of soil organic P pools and is highly correlated with soil P availability (Colvan et al., 2001; Saha et al., 2008). Both PAB and PSB play crucial roles in the fate of microbial biomass P, as stated above. However, most current studies focus on one aspect, either phosphorus solubilization or phosphorus aggregation by bacterial strains, while few address both aspects simultaneously. Research exploring both phosphorus solubilization and aggregation by strains as well as their potential agricultural applications remains limited. Therefore, we hypothesize that natural bacterial strains exist that simultaneously possess dual functions of efficient phosphorus solubilization and phosphorus accumulation. The synergistic interaction between these strains’ phosphorus solubilization and accumulation capabilities jointly regulates the availability of phosphorus in soil. Based on this, the research objectives: (1) Screen multifunctional strains capable of both phosphorus solubilization and phosphorus accumulation; (2) Quantitatively evaluate the phosphorus solubilization and accumulation capabilities of selected strains; (3) Reveal the intrinsic relationship and mechanisms between phosphorus solubilization and accumulation; (4) Assess their potential value for agricultural applications. We believe that this study provides valuable insights into sustainable phosphorus management in agriculture and may have future applications in various agricultural settings.

## 2 Materials and methods

### 2.1 Materials

Water samples were collected from South Lake in Changchun City, Jilin Province (125.31° E, 43.85° N). The supernatant was continuously diluted into solutions with different concentration gradients of 10^−4^–10^−8^. These solutions were then coated onto Luria Bertani (LB) solid medium and incubated in a constant temperature incubator at 30 °C for 8 to 24 h. Single colonies inoculated into 10 mL of LB liquid medium. The cultures were then placed in a shaker at 150 rpm and 30 °C until reaching an optical density (OD600) of 0.8. Next, 2.5 μL of the culture was pipetted onto the center of the modified inorganic phosphorus solid medium; furthermore, inoculated into the solid isolation medium of B. polyphosphoriae (PAM) using the plate scribing method with a sterile inoculation ring dipped in the LB bacterial solution. Finally, all plates were inverted in an incubator at a constant temperature of 30 °C and incubated for 2–3 days. Three replicates were prepared for each experimental group.

### 2.2 Identification of phosphorus-solubilizing polyphosphate bacteria

Granule staining was performed to investigate the presence of polyphosphate (Poly-P) according to the methods described in previous literature (Wan et al., 2017). In addition, the samples were subjected to 16S rRNA sequencing and phylogenetic analysis as outlined in the literature and were identified using the Gram staining method (Buchanan and Gibbons, 1974). The isolated strains and all selected colonies were numbered and stored at 4 °C for later use.

### 2.3 16S rRNA gene sequencing

The bacterial genomic DNA extraction kit from Beijing Novogene Co., Ltd. was used to extract the strain’s DNA. The extracted DNA served as a template for PCR amplification using the bacterial universal primers 27F (5′-AGAGTTTGATCCTGGCTCAG-3′) and 1492R (5′-GGTTACCTTGTTACGACTT-3′). The amplification system comprised the following components: 1 μL of DNA template, 0.5 μL of primer 27F, 0.5 μL of primer 1492R, 12.5 μL of 2 × TaqMix, and 10.5 μL of ddH2O. The PCR procedure is as follows: 94 °C for 4 min, followed by 30 cycles of 94 °C for 30 s, 55 °C for 30 s, 72 °C for 2 min, concluding with 72 °C for 10 min. The amplified products were sequenced bidirectionally by Beijing Novogene Co., Ltd. The 16SrDNA sequences were spliced together using Contig Express and subsequently searched and analyzed in the GenBank and NCBI databases. A phylogenetic tree was constructed using the neighbor-joining method in MEGA version 7.0, and the robustness of the tree was evaluated through bootstrap analyses based on 1,000 replications (Felsenstein, 1985).

### 2.4 Phosphorus dissolving capacity and pH determination experiments

The selected strains were cultured in LB liquid medium at 30 °C, 180 rpm for 24 h (v/v), reaching OD600 = 0.8. Subsequently, 1 mL of the activated strain was transferred into 100 mL of sterilized NBRIP liquid medium, with tricalcium phosphate as the sole phosphorus source. The cultures were then incubated under aerobic and anaerobic conditions, respectively, in an incubation shaker at 150 rpm and 30 °C for 96 h, with 3 replications per strain, the supernatant was collected to measure the amount of P solubilized and the pH for each strain. The concentration of AP was determined using the molybdenum blue colorimetric assay method (Waterlot, 2018). Strains demonstrating high P solubilization capacity were stored at 4 °C for use in subsequent studies.

### 2.5 Phosphorus polymerizing capacity experiments

Seven different phosphorus concentrations established: (1) 8 g L^−1^, (2) 4 g L^−1^, (3) 1 g L^−1^, (4) 0.5 g L^−1^, (5) 0.1 g L^−1^, (6) 0.01 g L^−1^, and (7) 0.001 g L^−1^. A single colony was inoculated into 10 mL of LB liquid medium. The colony at 30 °C and 150 rpm for cultivation reached OD600 of 0.8, it was inoculated into 7 phosphorus concentrations of PAM medium at a 1% inoculum level and cultivated for 6 days. Three replicates were established for each group.

(1) The PAM medium was incubated in a 150 rpm shaker at 30 °C. 2 mL was used to assess the growth of the strains at OD600, while the 2 mL was centrifuged to measure the phosphorus content in the supernatant at a wavelength of 660 nm.
(2) 10 mL of fermentation broth, the supernatant was discarded, wet weight of the organisms was measured. Next, 10 mL of cell lysate was added to resuspend the organisms, and the organisms were ultrasonically crushed on ice. The phosphorus content in the organisms was determined using the molybdenum blue colorimetric assay (Waterlot, 2018). Finally, the change in phosphorus content per unit of organism in the medium at different phosphorus concentrations was calculated.

### 2.6 Functional characterization of phosphorus-aggregating bacteria under conditions of high and low phosphorus medium exchange

(1) Phosphorus-releasing capacity of strains in low-phosphorus media.

When the concentration of bacteria in LB medium reached an OD_600_ of 0.8, it was inoculated into 250 mL of PAM medium containing 4 g L^−1^ of phosphorus at a 1% inoculum. It was then incubated for 3 days at 30 °C in a 150 rpm shaker, then centrifuged at 8,000 rpm for 10 min. The supernatant was poured off and added to 250 mL of 0.1 g L^−1^ phosphorus and phosphorus-free PAM medium. Aerobic and anaerobic cultures were conducted separately, with 4 mL of fermentation broth taken at 1-day intervals for phosphorus determination.

(2) Phosphorus polymerizing capacity of strains in high-phosphorus media.

250 mL of 0.1 g L^−1^ phosphorus-concentrated PAM medium was added to a 30 °C 150 rpm shaker for 4 days and centrifuged. The supernatant was poured off and 250 mL of 4 g L^−1^ PAM medium was added, mixing until there was no precipitation of bacterial organisms. Finally, 4 mL of the fermentation broth was taken each day to determine the phosphorus content.

### 2.7 Determination of enzyme activities

(1) The determination of Exopolyphosphatase (PPX) activity was conducted based on the references (Cox et al., 1981; Lee et al., 2006). The reaction was conducted at 30 °Cafter adding 50 μL of crude extracts to a reaction mixture containing 0.5 M Tris–HCl buffer (pH 7.4), 5 mM MgCl_2_, and 2.5 mM p-nitrophenyl phosphate. After a 45-min incubation, 2 mL of 0.5 mol/L KOH was added to terminate the reaction, followed by measuring the absorbance at 405 nm. Specific PPX activity was defined as the production of μmol *p*-nitrophenol/(min·mg·protein).
(2) The assay of polyphosphate (poly-P) utilization was used to determine the polyphosphate kinase (PPK) activity (Robinson and Wood, 1986; Van Groenestijn et al., 1989). The reaction was initiated by adding ADP, resulting in a final concentration of 1 mM. The produced NADPH was measured spectrophotometrically at 340 nm (Zhang et al., 2000). Specific PPK activity was determined as the production of μmol NADPH/(min·mg·protein).
(3) Glucose dehydrogenase enzyme (GDH) assay. GDH enzyme activity was measured using a chromogenic assay that involved 2,6 dichlorophenolindophenol (DCIP; Fisher Scientific) and phenazine methosulfate (PMS; Sigma-Aldrich), as described by Matsushita and Ameyama (1982). The enzyme activity was measured as the initial reduction rate of DCIP, monitored using a DU800 UV/visible spectrophotometer (Beckman Coulter) at 600 nm. Specific enzyme activity was expressed in units per milligram of protein, with 1 unit defined as the reduction of 1 mol of DCIP per minute.

### 2.8 Transcriptomic measurement and analysis

Two groups of samples were prepared: PAM-G (J4G, 4 g L^−1^ phosphorus concentration, glucose, and bacteria) and PAM-G-Ca_3_(PO_4_)_2_ (JCaG, 4 g L^−1^ phosphorus concentration, Ca_3_(PO_4_)_2_, glucose, and bacteria). The samples were designated as J4G and JCaG, respectively. Each sample was frozen in liquid nitrogen, and three biological replicates were collected. Total RNA was extracted from the samples using TRIzol reagent, and RNA integrity was assessed using the RNA Nano 6,000 Assay Kit on the Bioanalyzer 2,100 system (Agilent Technologies Inc., Santa Clara, CA, United States). The cDNA library was sequenced using Illumina technology at Beijing Novogene Bioinformatics Technology Co., Ltd. (Beijing, China). An index of the reference genome was built using Hisat2 v2.0.5, and paired-end clean reads were aligned to the reference genome using the same software. FeatureCounts v1.5.0-p3 was used to count the number of reads mapped to each gene. The FPKM of each gene was calculated based on the gene length and the number of reads mapped to it (for DESeq2 with biological replicates). Differential expression analysis of two conditions/groups (with two biological replicates per condition) was performed using the DESeq2 R package (version 1.20.0). Gene Ontology (GO) and Kyoto Encyclopedia of Genes and Genomes (KEGG) tools were used to analyze the differentially expressed genes (DEGs).

### 2.9 Determination of gene expression levels by quantitative real-time PCR

RNA was extracted using Trizol (TransGen, Beijing) and reverse transcribed using the PerfectStart* Uni RT&qPCR Kit (TransGen, Beijing). Fluorescent quantitative PCR primers were designed based on the specific sequences of each gene (Table S1). 16S rRNA was used as the internal reference gene, and fuorescent quantitative PCR was performed using the TransGen SYBR Premix Ex Taq II kit (TransGen, Beijing, China) with an ABI StepOne Plus real-time fuorescent quantitative PCR instrument (ABI, USA, model 7,300). The 20-μl reaction system contained 2 μL of cDNA and 10 μL of SYBR. The PCR program consisted of 94 °C for 30 s (pre-denaturation), 94 °C for 5 s (denaturation), and 55 °C for 15 s (annealing-extension) for a total of 45 cycles. Data were calculated using the 2^−ΔΔct^ algorithm for relative expression (Livak and Schmittgen, 2001). All experiments were conducted in triplicate. Statistix (v 8.1) software was used to analyze the significance of the data.

### 2.10 Experimental design and plant growth conditions

Rice seeds were surface-sterilized with 20% H_2_O_2_ for 20 min, then rinsed 3–5 times with sterile distilled water and dried on a sterile plate. The seeds were germinated for 2 days at 25 °C. For each treatment, eight rice seedlings were planted in each pot 1 day after the bacteria were inoculated, marking the planting day as the first day. The pots were then placed in a greenhouse under a day/night regimen of 10/14 h at temperatures of 25/28 °C, with a photon flux density of 150 mmol m^−2^ s^−1^. The pots were watered daily to maintain soil moisture at 24.8%. After being cultivated under sunlight for 15 days, the rice seedlings grew to approximately 10 cm in height.

(1) 500 mL paper cups were prepared, and 0.1 g L^−1^, 0.5 g L^−1^, and 4 g L^−1^ P gradients were set up using Na_2_HPO_4_ and KH_2_PO_4_ in a 1/2 Hoagland nutrient solution. Three treatment groups were established: Group A transferred rice seedlings with uniform growth to the above nutritional solution. Group B transferred the organisms to a 1/2 Hoagland nutrient solution without P after 4 days of aerobic incubation with strain NHP4a-2 added to the nutrient solution at each P gradient. Similarly, Group C transferred the organisms to a 1/2 Hoagland nutrient solution without P after 4 days of aerobic incubation with strain NHP4b-2 added to the nutrient solution at each P gradient. CK1 consisted of 1/2 Hoagland nutrient solution without P, while CK2 contained 1/2 Hoagland nutrient solution with normal levels of P (Supplementary Figure S3). Five replicates of each treatment were established.
(2) In addition, plastic cups were prepared and filled with sand in the cups. Four treatments were set up as follows: CK1: (no Ca_3_ (PO_4_)_2_), CK2: (Ca_3_ (PO_4_)_2_), A: (NHP4a-2 with Ca_3_ (PO_4_)_2_), and B: (NHP4b-2 with Ca_3_ (PO_4_)_2_). Germinated rice seeds were placed in the center of each hole, with 8 seeds per hole. Two strains of bacteria were cultured in LB liquid medium until an OD600 of approximately 0.8 was reached. Then, 3 mL of the bacterial solution was sucked up and added to the germinated rice seeds, along with 2.5 g of Ca_3_ (PO_4_)_2_ (Supplementary Figure S4). Five replicates were established for each treatment.

### 2.11 Measurement of rice plant indexes

The rice seedlings were scanned using a root scanner (Epson Expression V800 photo) and analyzed for several root traits: root length (cm), root mean diameter (cm), root volume (cm^3^), root surface area (cm^2^), root projected area (cm^2^), and the number of root tips. These measurements were obtained using a plant root system analyzer (WinRHIZO). The roots were gently spread on a flat tray filled with a shallow layer of water and then placed into an Epson flatbed scanner with a resolution of 300 dpi. The above morphological root traits in response to the co-application of PolyB-PSBCs were automatically quantified using WinRhizo software (Regent Instruments Inc., Québec, QC, Canada). The plant phosphorus (Pi) contents were determined spectrophotometrically using the molybdate blue method (Khourchi et al., 2022).

### 2.12 Statistical analysis

Each experiment was repeated three times. The means and standard deviations for each treatment were calculated and presented. The 16S rRNA sequences were compared using the BLAST program, and a phylogenetic tree was constructed with MEGA 7.0 software. Charts were created using Origin 2022 and Office 365.

## 3 Results

### 3.1 Screening of phosphorus-solubilizing polyphosphate bacteria

**Figure 1:**
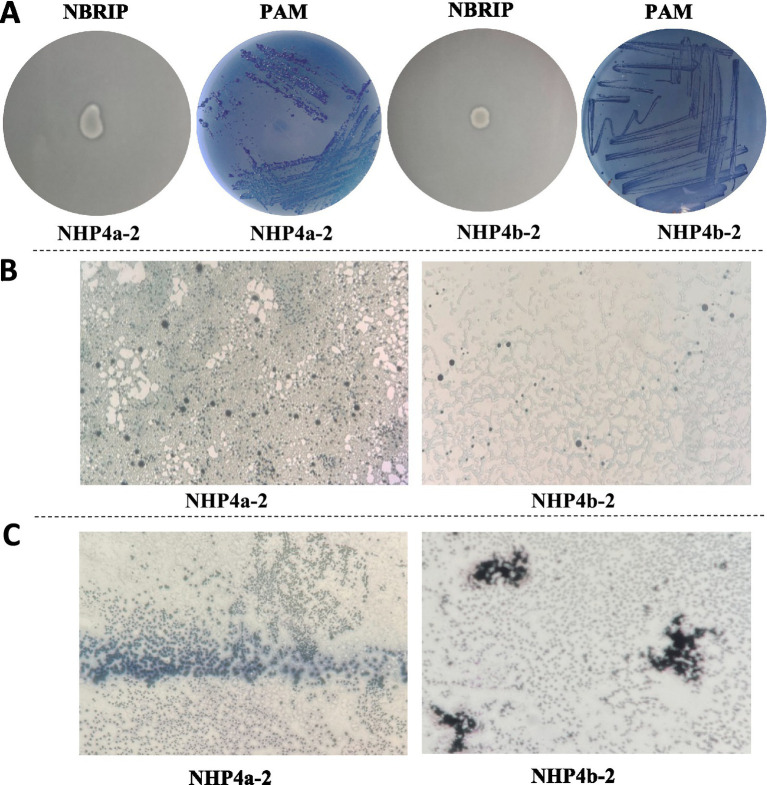
Screening and identification of phosphorus-solubilizing polyphosphate bacteria. **(A)** Screening of strains with phosphorus solubilizing and phosphorus accumulating abilities. **(B)** Metachromatic granule staining results of strains (40×), blue granules indicate azo granules. **(C)** Results of PHB staining of strains (40×), blue-black granules indicate PHB granules.

Using the concentration gradient dilution method, the NBRIP medium and PAM solid isolation medium were subjected to inverted incubation for 2 to 3 days. It was observed that two strains of bacteria exhibited clear, transparent circles on the modified NBRIP solid medium. This phenomenon may be attributed to the phospholytic bacteria, which produce significant amounts of organic acids during their growth process. These acids can break down the calcium phosphate in the medium, thus forming a distinct transparent zone around the bacteria (Figure 1A). However, the specific types of organic acids produced require further verification. The preliminary results indicated that both strains had the ability to solubilize phosphorus. A significant lightening of the blue color was observed in the PAM solid isolation medium (Figure 1A), indicating that the strains can remove phosphorus and absorb excess phosphate from the surrounding environment into their body. This preliminary finding indicates that both strains possess the functions of phosphorus solubilization and polyphosphate utilization. These strains were named NHP4a-2 and NHP4b-2, respectively. This qualitative method enhances the screening accuracy, saves human and material resources, and serves as a more efficient approach for isolating phosphorus solubilization and polyphosphate bacteria from the environment.

*E. coli* was used as a blank control (CK), while NHP4a-2 and NHP4b-2 were stained with heterochromatic particles. Analysis of the staining results showed black particles in both strains (Figure 1B), indicating that they were capable of producing polypolyphosphate particles. The PHB staining test revealed the presence of blue-black particles in both strains (Figure 1C), further confirming that both strains had polyphosphate production capabilities.

### 3.2 Identification of phosphorus-solubilizing polyphosphate bacteria

**Figure 2:**
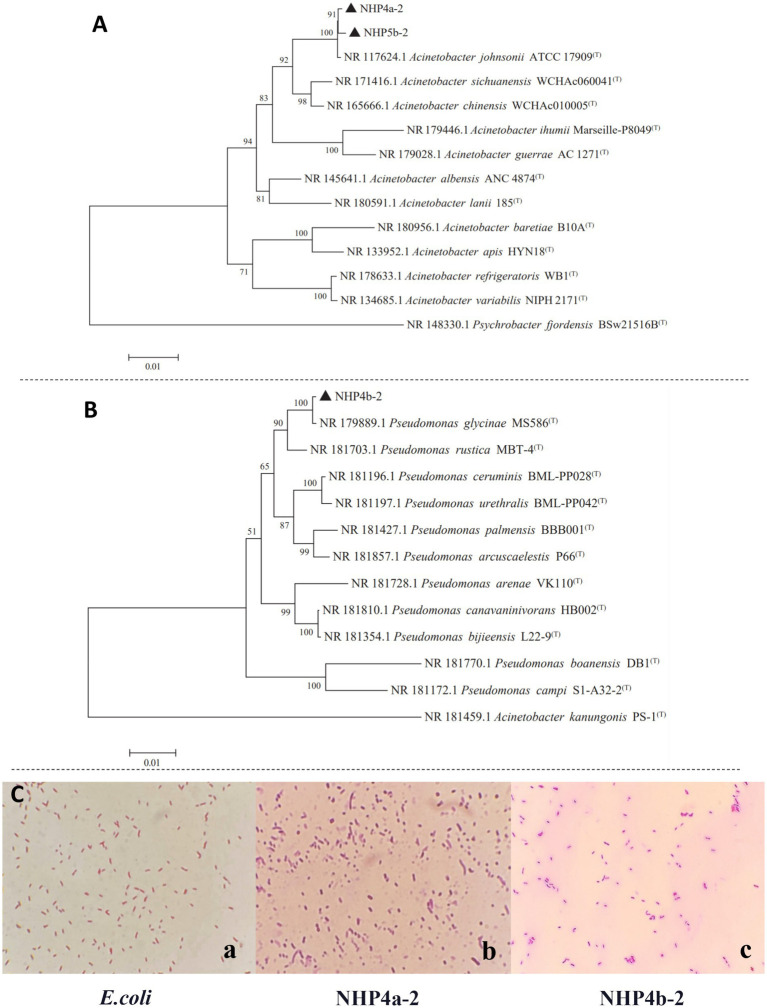
Construction of a phylogenetic tree of strain 16S rDNA sequences and gram staining **(A,B)** phylogenetic tree of strain NHP4a-2. **(C)** Gram staining microscopic images of strains NHP4a-2 (b) and NHP4b-2 (c) (100×), *Escherichia coli* as a blank control (a).

The nucleotide sequences of the 16S rRNA genes from the two selected strains were amplified by PCR and sequenced. These sequences were then compared to those in the NCBI database using BLAST. A phylogenetic tree was constructed using the neighbor-joining method in MEGA 7 software. Strain NHP4a-2 was found to be most closely related to Acinetobacter johnsonii ATCC 17909, with 99.47% homology (Figure 2A), confirming that strain NHP4a-2 belonged to the *Acinetobacter* genus. Strain NHP4b-2 was most closely related to Pseudomonas glycinae MS586, with 99.80% homology, indicating it belonged to the *Pseudomonas* genus (Figure 2B). Gram staining results indicated that both NHP4a-2 and NHP4b-2 organisms were stained red (Figure 2C), suggesting that they are Gram-negative bacteria.

### 3.3 Determination of phosphorus solubilizing capacity of strains

**Figure 3:**
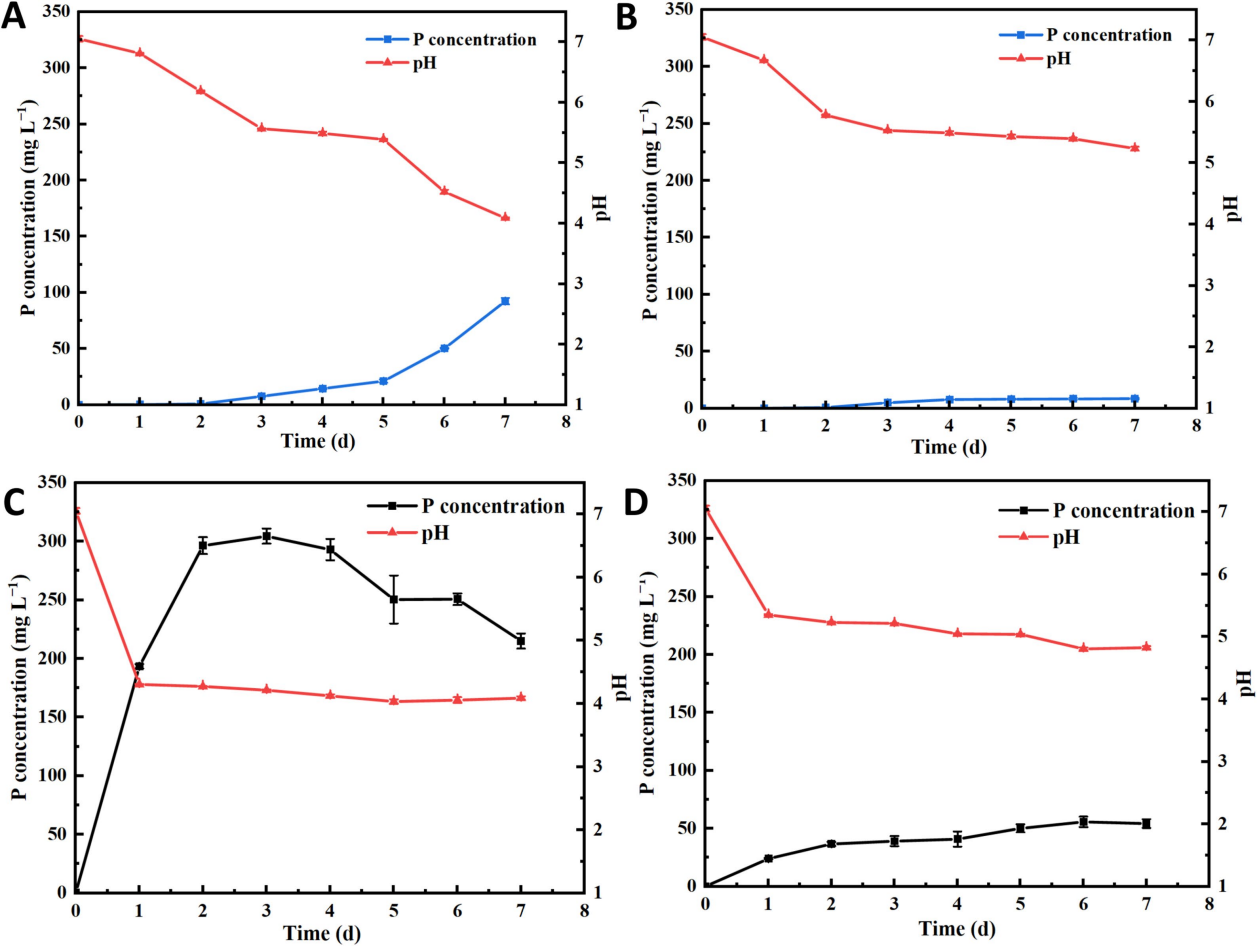
Phosphorus solubilization and medium pH changes of strains under aerobic anaerobic conditions. **(A)** Dissolved phosphorus and pH of NHP4a-2 under aerobic conditions. **(B)** Dissolved phosphorus and pH of NHP4a-2 under anaerobic conditions. **(C)** Dissolved phosphorus and pH of NHP4b-2 under aerobic conditions. **(D)** Dissolved phosphorus in NHP4b-2 under anaerobic conditions and pH.

The phosphorus solubilizing capacity of strains NHP4a-2 and NHP4b-2 was determined under aerobic and anaerobic conditions, respectively. Strain NHP4a-2 exhibited a maximum phosphorus solubilizing capacity of 92.47 mg/L on the 7th day under aerobic conditions, with the pH dropping to 4.1 (Figure 3A). Under anaerobic conditions, the maximum phosphorus solubilizing capacity reached 8.42 mg L^−1^ on the 7th day, with a pH of 5.26 (Figure 3B). Strain NHP4b-2, on the other hand, achieved a maximum phosphorus solubilizing capacity of 310.00 mg L^−1^ on the 3rd day under aerobic conditions, with the pH at 4.2 (Figure 3C). The highest amount of dissolved phosphorus reached 53.6 mg L^−1^ on the 7th day under anaerobic conditions, with a pH of 4.81 (Figure 3D). A comparison of the two strains showed that strain NHP4b-2 had a stronger phosphorus solubilizing ability than NHP4a-2. It was also found that the phosphorus solubilizing ability was stronger under aerobic conditions, indicating that both strains are better adapted to aerobic environments for this function. In addition, as time increased, the pH value of the medium gradually decreased while the amount of dissolved phosphorus increased.

### 3.4 Determination of phosphorus polyphosphate capacity of strains

**Figure 4:**
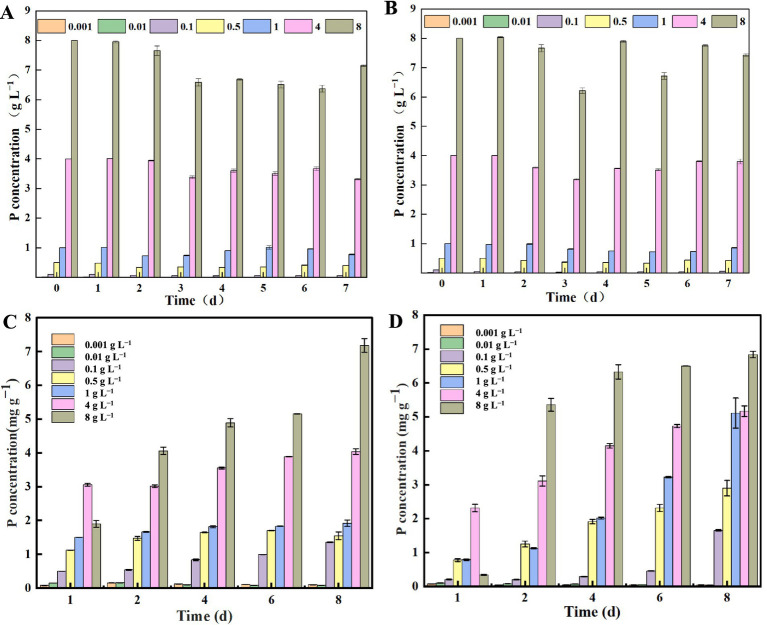
Determination of phosphorus polyphosphate capacity of strains. **(A)** Changes in the phosphorus concentration of *NHP4a-2* in the supernatants of media with different phosphorus concentrations. **(B)** Changes in the phosphorus concentration of *NHP4b-2* in the supernatants of media with different phosphorus concentrations. **(C)** Changes in phosphorus content per unit of bacteria in NHP4a-2 in different phosphorus media. **(D)** Changes in phosphorus content per unit of bacteria in NHP4b-2 in different phosphorus media.

Seven different phosphorus concentration gradients of PAM medium were set up: 0.001 g L^−1^, 0.01 g L^−1^, 0.1 g L^−1^, 0.5 g L^−1^, 1 g L^−1^, 4 g L^−1^, and 8 g L^−1^, respectively. The phosphorus aggregation capacity of strains NHP4a-2 and NHP4b-2 was measured over a period of 7 days. At 0.001 g L^−1^ and 0.01 g L^−1^, the phosphorus concentrations were likely too low, resulting in only a slight increase in bacterial volume and slow growth. At 0.1 g L^−1^ phosphorus concentration, strain NHP4a-2 reached a maximum phosphorus uptake of 47.457 mg L^−1^ on day 4, while strain NHP4b-2 reached 70.967 mg L^−1^ on day 3 (Figures 4A,B). At a phosphorus concentration of 0.5 g L^−1^, strain NHP4a-2 reached a maximum phosphorus uptake of 159.40 mg L^−1^ on day 5, while strain NHP4b-2 reached 165.063 mg L^−1^ on day 3 (Figures 4A,B). At a phosphorus concentration of 1 g L^−1^, strain NHP4a-2 reached its highest phosphorus uptake of 272.17 mg L^−1^ on day 2, whereas strain NHP4b-2 reached its highest phosphorus uptake of 281.063 mg L^−1^ on day 5 (Figures 4A,B). At 4 g L^−1^ phosphorus concentration, both strains NHP4a-2 and NHP4b-2 reached their highest phosphorus uptake on day 3 (Figures 4A,B). At 8 g L^−1^ phosphorus concentration, strain NHP4a-2 reached its highest phosphorus uptake on day 6, while strain NHP4b-2 reached its highest phosphorus uptake of 1780.907 mg L^−1^ on day 2 (Figures 4A,B). The phosphorus uptake capacity of strains NHP4a-2 and NHP4b-2 gradually increased with the increase of phosphorus concentrations in the medium.

Changes in phosphorus content per unit of bacterial biomass were also determined during the incubation process. Phosphorus concentrations of 0.01 g L^−1^ and 0.001 g L^−1^ exhibited the highest phosphorus content on day 2 for both strains, likely due to insufficient phosphorus concentration for bacterial growth. Conversely, a phosphorus concentration of 0.1 g L^−1^ showed the highest phosphorus levels on day 8 for strains NHP4b-2 and NHP4a-2. In strain NHP4a-2, the 0.5 g L^−1^ phosphorus concentration peaked on day 6, while strain NHP4b-2 reached its maximum on day 8. At phosphorus concentrations of 1 g L^−1^ and 8 g L^−1^, both strains NHP4a-2 and NHP4b-2 reached their maximum on day 8 (Figures 4C,D). Consequently, the phosphorus content in strains NHP4a-2 and NHP4b-2 exhibited a gradient trend with an increasing number of days at phosphorus concentrations ranging from 0.1 g L^−1^ to 8 g L^−1^. Notably, the phosphorus concentration in strain NHP4b-2 was higher than that in strain NHP4a-2. The experimental results further confirmed that the phosphorus concentration in both strains, NHP4a-2 and NHP4b-2, increased with the initial phosphorus concentration in the medium and the increase in the incubation duration. This indicated that the ability to polymerize phosphorus in the bacteria also gradually improved.

**Figure 5:**
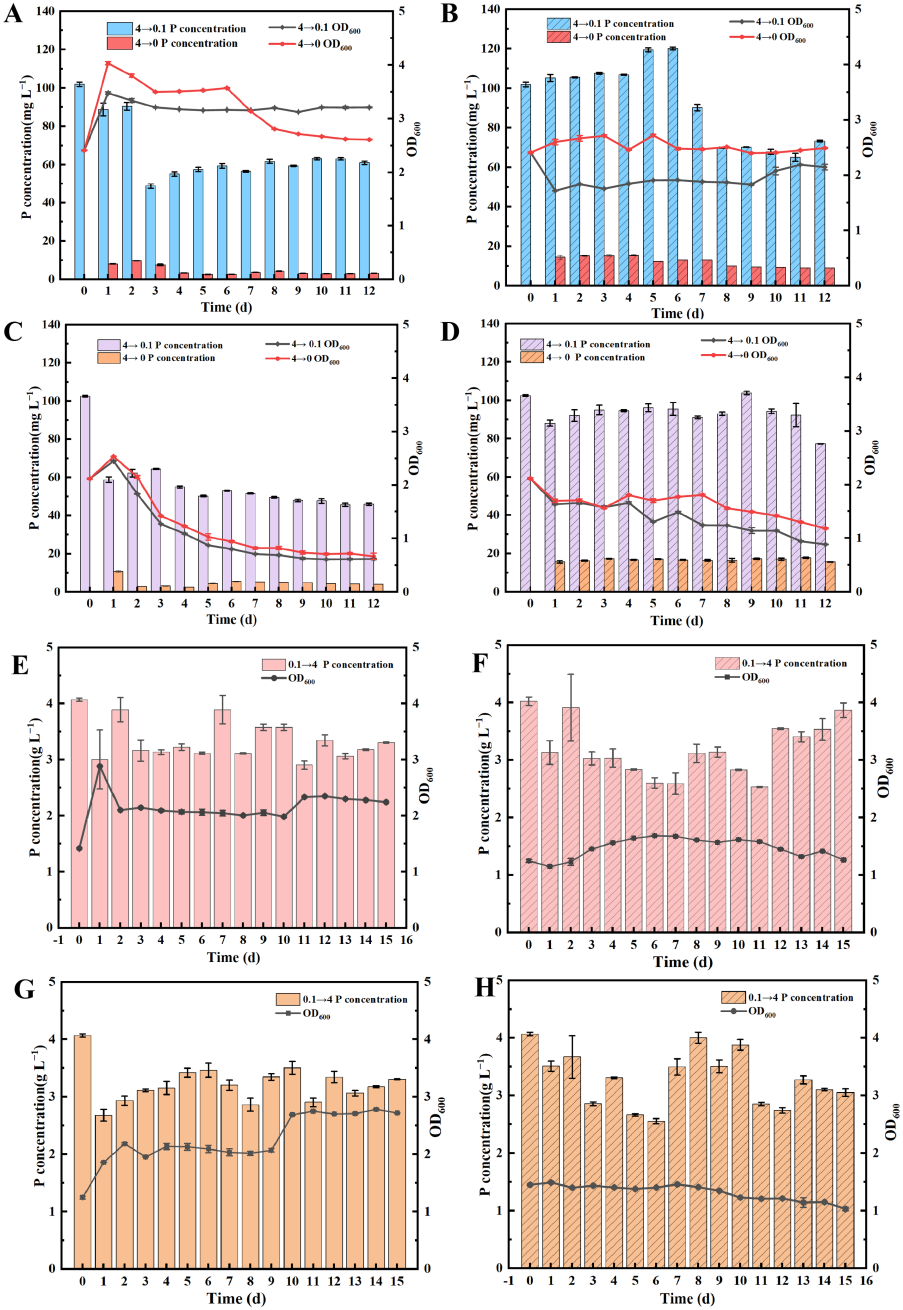
Functional analysis of phosphorus-polymerizing bacteria under high and low phosphorus medium exchange conditions. **(A)** Phosphorus release capacity of NHP4a-2 in low phosphorus medium under aerobic conditions. **(B)** Phosphorus release capacity of NHP4a-2 in low phosphorus medium under anaerobic conditions. **(C)** Phosphorus release capacity of NHP4b-2 in low phosphorus medium under aerobic conditions. **(D)** Phosphorus release capacity of NHP4b-2 in low phosphorus medium under anaerobic conditions. **(E)** NHP4a-2 phosphorus accumulation capacity under aerobic conditions under high phosphorus conditions. **(F)** NHP4a-2 phosphorus accumulation capacity under anaerobic conditions under high phosphorus conditions. **(G)** NHP4b-2 phosphorus accumulation capacity under aerobic conditions under high phosphorus conditions. **(H)** NHP4b-2 phosphorus accumulation capacity under anaerobic conditions under high phosphorus conditions.

In order to investigate the phosphorus release capability of polyphosphate bacteria in low-phosphorus medium, strains NHP4a-2 and NHP4b-2 were cultured in a 4 g L^−1^ phosphorus-concentrated PAM medium. They were then transferred to 0.1 g L^−1^ and phosphorus-free PAM mediums for further cultivation. Under aerobic conditions, neither NHP4a-2 nor NHP4b-2 released phosphorus. Instead, the phosphate from the medium was gradually absorbed into the body over time. The OD600 of both strains reached the maximum on day 1 and then gradually decreased and stabilized. (Figures 5A,C). This indicates that the strains find it difficult to release phosphorus under aerobic conditions, even with insufficient phosphorus requirements for growth. Under anaerobic conditions, at a phosphorus concentration of 0.1 g L^−1^, strain NHP4a-2 gradually released phosphorus with the increase of the number of days. However, its release ability began to decline after day 7. In contrast, strain NHP4b-2 exhibited the highest phosphorus release capacity on day 9. In phosphorus-free PAM medium, strain NHP4a-2 gradually released phosphorus from days 1–4, after which its ability to release phosphorus decreased. Similarly, strain NHP4b-2 gradually released phosphorus from days 1–3 before its release ability declined. Both strains OD600 exhibited slow growth under anaerobic conditions (Figures 5B,D). In summary, neither strains NHP4a-2 nor NHP4b-2 released phosphate under aerobic conditions. However, under anaerobic conditions, phosphorus-aggregating organisms cultured in high-phosphorus medium were transferred to low-phosphorus medium to promote bacterial growth and facilitate phosphorus release into the surrounding environment. The ability to release phosphorus was stronger in phosphorus-free medium. In addition, strain NHP4b-2 demonstrated a stronger ability to accumulate polyphosphate compared to strain NHP4a-2.

Strains NHP4a-2 and NHP4b-2 were cultured in a 0.1 g L^−1^ phosphorus-concentrated PAM medium and then transferred to a 4 g L^−1^ PAM medium to investigate the phosphorus aggregation ability of phosphorus-starved organisms under high phosphorus conditions. Under aerobic conditions, both strains began to aggregate phosphorus on day 1, after which the aggregation leveled off. The phosphorus concentration in the supernatant reached approximately 3.34 g L^−1^ for NHP4a-2 and 3.02 g L^−1^ for NHP4b-2 (Figures 5E,G). Organisms from both strains cultured in low-phosphorus conditions under anaerobic conditions were then transferred to high-phosphorus medium, resulting in a slower release of phosphate and slower growth (Figures 5F,H). The results indicated that strain NHP4b-2 exhibited a stronger ability to polyphosphate compared to NHP4a-2 under aerobic conditions.

### 3.5 Analysis of enzyme activities in the process of phosphorus solubilization and polyphosphate polymerization by strains

**Figure 6:**
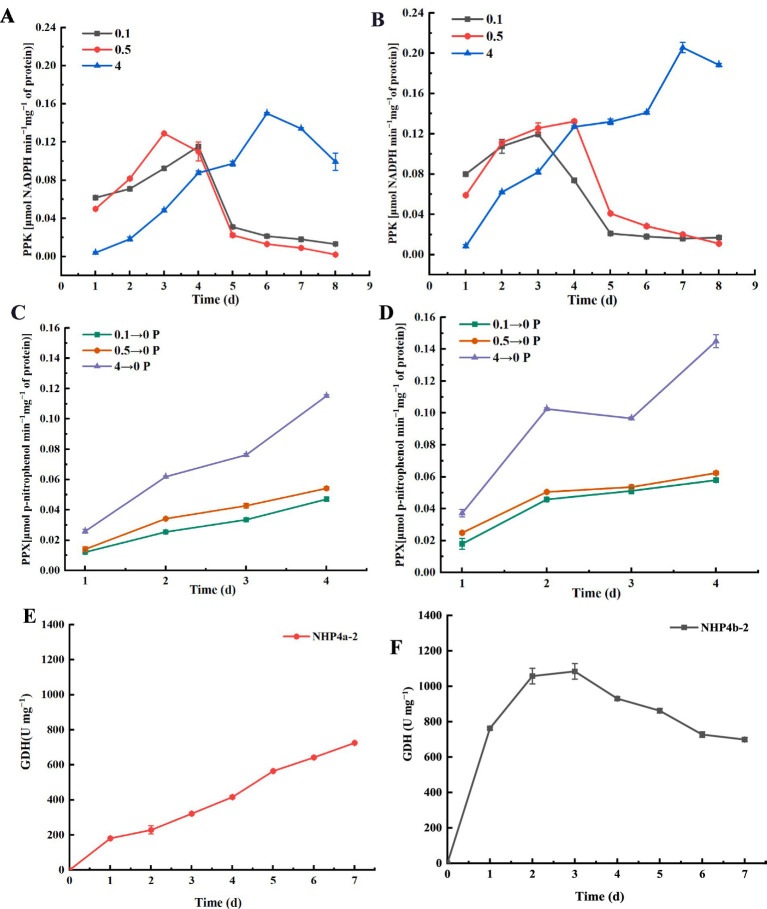
Analysis of enzyme activities. **(A)** Strain NHP4a-2 polyphosphate kinase activity (PPK). **(B)** Strain NHP4b-2 polyphosphate kinase activity (PPK). **(C)** Polyphosphate esterase activity of the NHP4a-2 (PPX). **(D)** Polyphosphate esterase activity of the NHP4b-2 (PPX). **(E)** Glucose dehydrogenase activity (GDH) of the NHP4a-2. **(F)** Glucose dehydrogenase activity (GDH) of the NHP4b-2.

PPK and PPX jointly regulate the synthesis and catabolism of polyphosphate in bacteria. The results indicated that PPK activity initially increased and then decreased with the increase in incubation time. However, the increase in PPK activity lasted the longest at a phosphorus concentration of 4 g L^−1^. We hypothesized that higher phosphorus concentrations would promote the *in vivo* synthesis of poly-P, with PPK activity peaking at 4 g L^−1^ phosphorus concentration (Figures 6A,B). The PPX activity of both bacterial strains increased with the increase in the number of days, reaching a maximum on day 4. This increase is attributed to bacterial growth (Figures 6C,D), as PPX catalyzes the release of intracellularly stored poly-P into phosphate *in vitro*. Strain NHP4a-2 exhibited the highest GDH enzyme activity on day 7, while strain NHP4b-2 peaked on day 3, followed by a gradual decrease and stabilization (Figures 6E,F). The GDH activity and phosphorus solubilizing capacity of the two bacterial strains showed a consistent pattern, with the GDH activity of strain NHP4b-2 being higher than that of NHP4a-2.

### 3.6 Differential analysis of the expression of phosphorus solubilizing and polyphosphorus genes in strains

The previous study found that the strains were all in the polyphosphate state at 18 h for PAM-G (J4G) and in the phosphorus-solubilizing state for PAM-G-Ca3(PO4)2 (JCaG) (Supplementary Figures S1, S2). Therefore, 18 h was chosen as a sampling point to analyze the differential gene expression between the phosphorus solubilizing and polyphosphate functions.

**Figure 7:**
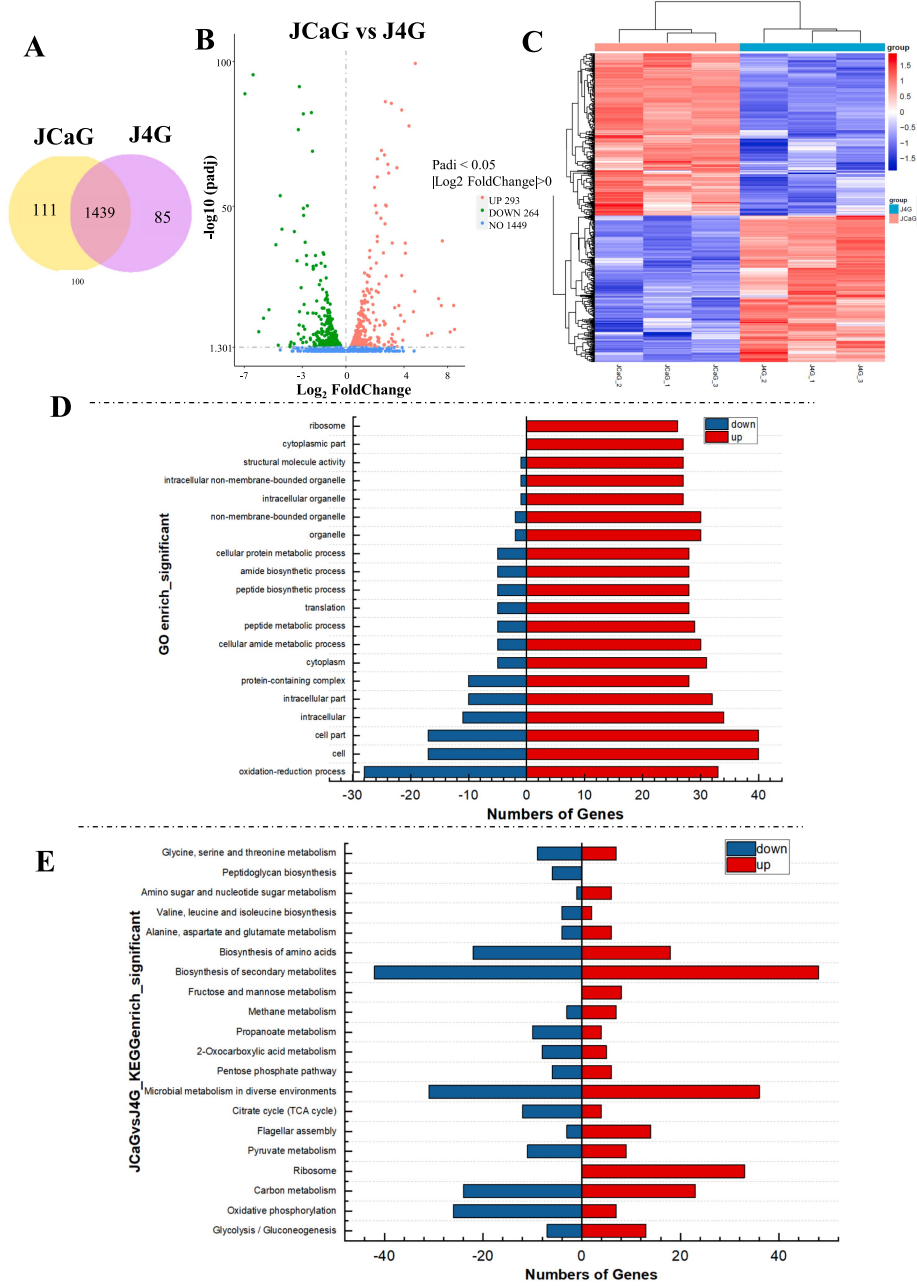
Differential transcriptome analysis of phosphorus solubilization and phosphorus accumulation in bacterial strains. **(A)** Venn diagram of co-expressed and differentially expressed genes in JCaG and J4G treatment groups. **(B)** Volcano diagram of differentially expressed genes between JCaG and J4G treatment groups. **(C)** Heat map of differential gene clustering of JCaG vs. J4G treatment groups. **(D)** The 20 most significantly enriched GO enrichment of differentially expressed genes between the JCaG and J4G treatment groups. **(E)** Top 20 most significant KEGG enrichment analyses of differentially expressed genes between JCaG and J4G treatment groups.

Differential gene expressions were analyzed in the JCaG and J4G treatment groups. A total of 1,439 genes were differentially expressed in both the JCaG and J4G treatment groups. Conversely, 111 genes were differentially expressed only in the JCaG treatment group, while 85 genes were differentially expressed only in the J4G control group (Figure 7A). In total, 1,449 DEGs were screened in the JCaG-treated group compared to the J4G control group, of which 293 DEGs were significantly upregulated and 264 DEGs were significantly downregulated (Figure 7B). The samples were clustered together based on genes exhibiting similar expression patterns, and the RNA-seq data were subjected to hierarchical clustering. The results indicated that transcript levels were significantly different between the J4G and JCaG treatment groups (Figure 7C).

In order to gain insight into the biological processes involved in the strain between phosphorus solubilization and phosphorus aggregation, we analyzed the enrichment of gene ontology (GO) terms for DEGs. The GO analysis revealed the top 20 enrichment categories for biological processes, cellular components, and molecular functions (Figure 7D, Supplementary Table S2). The significantly enriched GO terms for DEGs in the comparison between JCaG and J4G included “oxidation–reduction process,” “cell part,” “transmembrane transporter,” and “structural constituent of ribosome” (Figure 7C).

The KEGG analysis identified the 20 most significant pathways, revealing that most of the DEGs were enriched in glycolysis/gluconeogenesis, oxidative phosphorylation, carbon metabolism, ribosome, pyruvate metabolism, flagellar assembly, and the citrate cycle (TCA cycle) (Figure 7E, Supplementary Table S3). In addition, GO and KEGG results indicated that ATP and acids were supplied to the strain through ATP metabolism, oxidative phosphorylation, and glycolysis/glycolysis pathways, supporting its phospholysis function.

**Figure 8:**
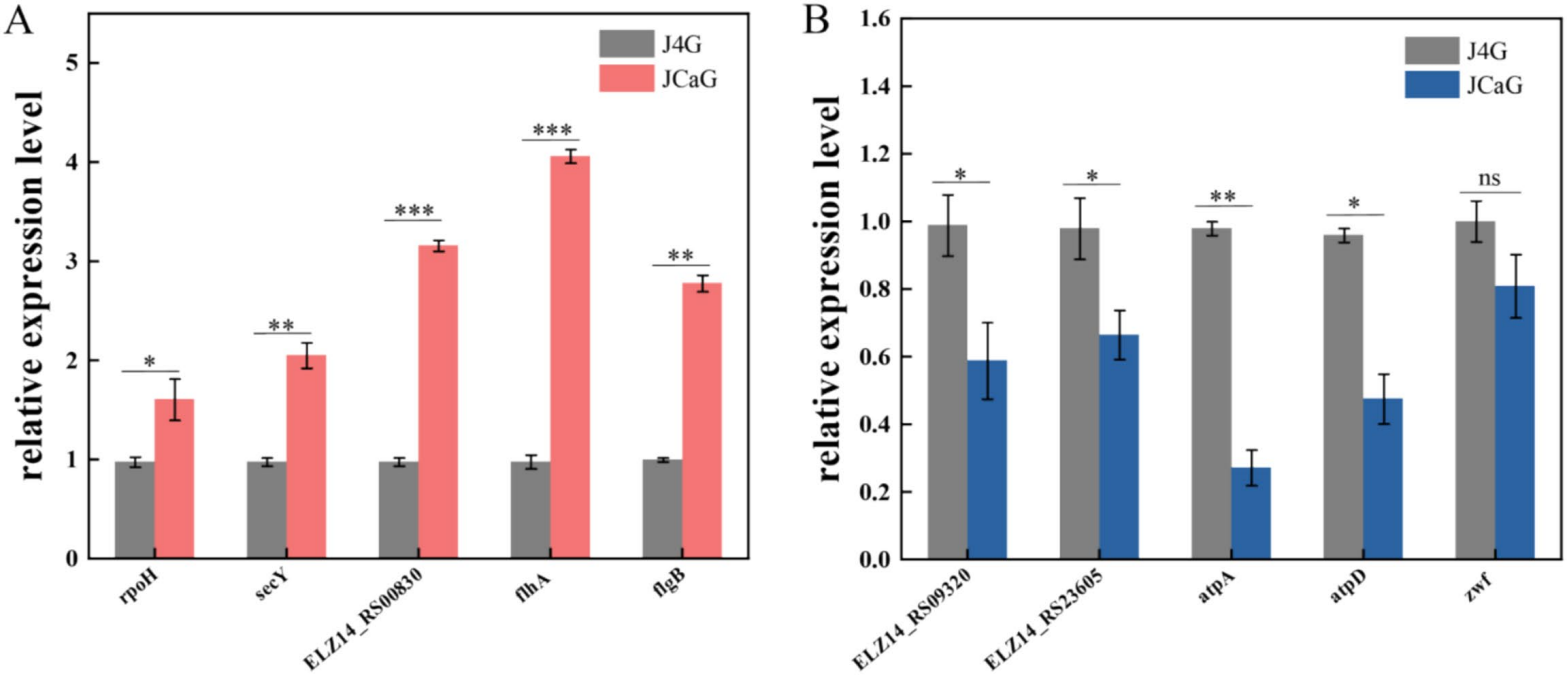
qRT-PCR detection of differentially expressed genes in phosphorus solubilization and phosphorus accumulation. **(A)** Relative expression levels of differentially up-regulated genes. **(B)** Relative expression levels of differentially down-regulated genes.

Finally, to validate our RNA-Seq data, quantitative RT-PCR (qRT-PCR) was performed on 10 DEGs (Figure 8, Supplementary Table S4). These genes exhibited similar expression patterns in both RNA-seq (FPKM) and qRT-PCR analyses, confirming the reproducibility and reliability of the RNA-seq data.

### 3.7 Effects of different states of strains on the growth and phosphorus content of rice seedlings

**Figure 9:**
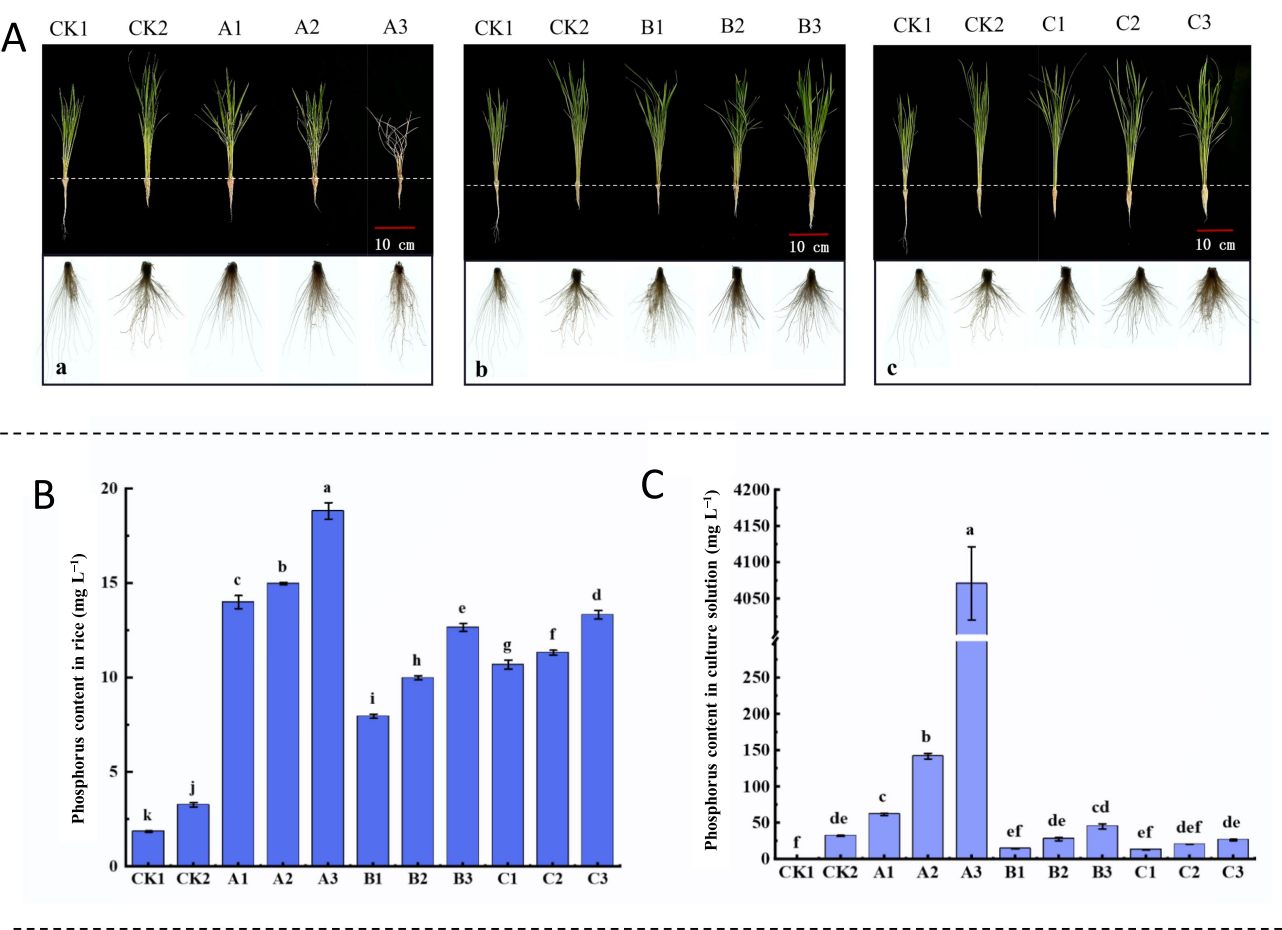
Effect of strains on the growth and phosphorus content of rice seedlings. **(A)** Effects of different states of strains on the growth of rice seedlings. **(B)** Phosphorus content in rice. **(C)** Phosphorus content in nutrient solution. Note: CK1, phosphorus-free nutrient solution. CK2, 1/2 Hoagland nutrient solution. A1, B1, C1: 0.1 g L P A2, B2, C2: 0.5 g L P; A3, B3, C3: 4 g L P. Group A: Sterile nutrient solution. Group B: NHP4a-2. Group C: NHP4b-2.

The strains NHP4a-2 and NHP4a-2 were aerobically cultured in PAM liquid medium with phosphorus concentrations of 0.1 g L^−1^, 0.5 g L^−1^, and 4 g L^−1^ for 4 days. Afterward, the organisms were transferred to a 1/2 Hoagland nutrient solution without phosphorus. The effects of growing the organisms in different phosphorus concentrations on the growth of rice seedlings were observed. The root lengths of seedlings in the CK1 group were significantly longer than those in the other treatment groups (Figure 9A). This may be attributed to the absence of phosphorus in the CK1 treatment group environment, which caused the rice seedlings to take up nutrients downward. The A3 treatment consisted of a nutrient solution with a phosphorus concentration of 4 g L^−1^, without the addition of strains. This concentration might have been too high and toxic for the growth of rice seedlings, leading to their death. The best growth phenotypes were observed in groups B and C, which also had a phosphorus concentration of 4 g L^−1^ (Figure 9A). This indicates that the amount of phosphorus absorbed and stored by the strains increased with the increase in phosphorus concentration in the medium. Consequently, more phosphorus can be released for plant growth when entering low-phosphorus conditions. Moreover, the effect of strain NHP4b-2 was better than that of strain NHP4a-2 across all treatment groups.

The phosphorus content in rice and its culture solution was determined under various treatments. The A3 treatment group exhibited the highest phosphorus content, followed by A2 and A1. In addition, the phosphorus levels in all three treatment groups were significantly higher than those in the blank control groups, CK2 and CK1. The phosphorus content of rice in the B3 treatment group was significantly higher than that in B2, B1, CK2, and CK1. Similarly, the phosphorus content of rice in the C3 treatment group was significantly higher than that in C2, C1, CK2, and CK1 (Figure 9B). After 35 days of incubation, the phosphorus content in the culture broth of rice seedlings was determined. It was found that the phosphorus content in the culture broth of the A3, B3, and C3 treatment groups was significantly higher than in C2, C1, CK2, and CK1. Moreover, the phosphorus content in the culture broth of the C treatment group was significantly lower than that of the B treatment group, which contrasted with the trend observed in the phosphorus content of the rice (Figure 9C). This discrepancy may be attributed to the release of phosphate by strain NHP4b-2 facilitating the uptake by rice. In summary, the phosphorus content in both rice seedlings and nutrient solutions across the three treatment groups aligned with the pattern of rice seedling phenotypic data. This further demonstrated that when bacteria absorb higher concentrations of phosphorus, more is stored in their bodies. Under low-phosphorus conditions, these bacteria release phosphate to support the growth of rice seedlings in hydroponics, with strain NHP4b-2 exhibiting a stronger ability to release phosphorus than strain NHP4a-2.

### 3.8 Effect of phosphorus solubilizing capacity of strains on the phenotype and phosphorus content of rice seedlings

**Figure 10:**
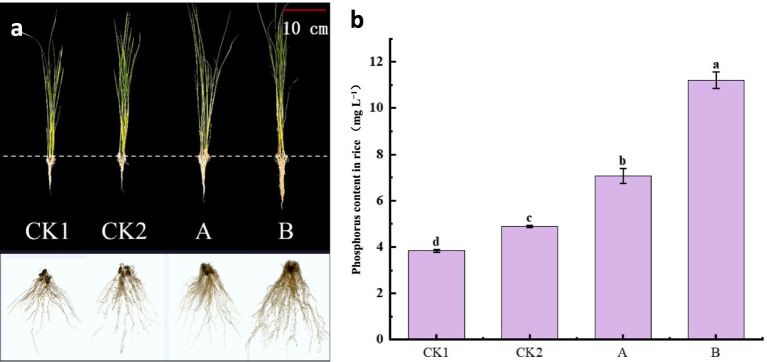
Effect of phosphorus solubilizing capacity of bacterial strains on the growth and phosphorus content of rice seedlings. **(A)** The growth of rice seedlings. **(B)** Phosphorus content of rice seedlings. CK1, without strain and Ca_3_(PO_4_)_2_. CK2, without strain + Ca_3_(PO_4_)_2_. **(A)** NHP4a-2 + Ca_3_(PO_4_)_2._ **(B)** NHP4b-2 + Ca_3_(PO_4_)_2_.

The impact of the phosphorus solubilizing capacity of the strain on the phenotype and phosphorus content of rice seedlings was observed after the addition of Ca_3_ (PO_4_)_2_ to fine sand planted with rice. Rice seedlings treated with strain NHP4b-2 exhibited significantly longer plant height, root length, leaf fresh weight, root fresh weight, root volume, root surface area, root projection area, and root tip number compared to those with strain NHP4a-2, followed by the CK2 and CK1 treatments (Figure 10A, Supplementary Table S5). This indicates that strain NHP4b-2 dissolved more insoluble Ca_3_(PO_4_)_2_ and exhibited a stronger phosphorus solubilizing capacity than strain NHP4a-2, thereby promoting the growth and development of the rice seedlings.

Exploring the effect of the strain’s phosphorus solubilizing capacity on the phosphorus content of rice seedlings revealed that the phosphorus content in rice treated with strain NHP4b-2 was significantly higher than that of the treatment groups of strains NHP4a-2, CK2, and CK1 (Figure 10B). Additionally, as shown in Figure 10A, the phenotypic changes in the rice seedlings further confirmed that the phosphorus-solubilizing capacity of strain NHP4b-2 was stronger than that of strain NHP4a-2, positively impacting the growth of the rice seedlings.

## 4 Discussion

PSB promotes plant growth by converting fixed phosphorus fertilizers in the soil into soluble phosphorus (Chen et al., 2022). Significant efforts have been made to isolate and characterize PSB from various soil environments (Devi et al., 2022; Fatima et al., 2021; Khan et al., 2007). Microorganisms with phosphorus-solubilizing function include Bacillus, Rhizobium, Flavobacterium, Acineobacter, and Agrobacterium, and others. (Fan et al., 2017; Cheng et al., 2023). In addition to phosphorus-solubilizing bacteria (PSB), the functional bacteria involved in the soil phosphorus cycle include a group of phosphorus-accumulating bacteria (PAB). PABs absorb excess phosphate from their surrounding environment into their body and store it in the form of poly-P particles in the bacteria. When phosphate levels in the environment are insufficient, they can decompose poly-P to release soluble phosphorus (Zhan et al., 2023). These microorganisms were also identified in various genera, including Flavobacterium, Pseudomonas (Neville et al., 2022), Acineobacter (Lv et al., 2022), and Bacillus (Wang et al., 2017). The concept of utilizing the P-accumulating characteristics of microorganisms for managing agricultural water environments has been proposed. However, it still lacks experimental validation (Saia et al., 2021). In this study, two strains exhibiting phosphorus solubilizing and phosphorus polymerizing functions were preliminary identified (Figure 1). In addition, 16S rDNA identification and phylogenetic tree analyses revealed that these two bacterial strains were Johne’s lactobacillus (NHP4a-2) and *Pseudomonas aeruginosa* (NHP4b-2), respectively (Figure 2). Since Johne’s lactobacillus is well-studied for its phosphorus-collecting functions, NHP4a-2 was selected as the reference strain to focus on the phosphorus solubilizing and phosphorus polymerizing properties of Pseudomonas NHP4b-2 and its potential application.

Tricalcium phosphate is the main form of inorganic phosphate precipitation. PSB can convert insoluble tricalcium phosphate into soluble phosphorus (Whitelaw et al., 1999). Wei et al. (2018) demonstrated that PSB can convert insoluble phosphate into a soluble form, this process is likely associated with the production of organic acids (OAs) and redox-activated metals, etc. OAs is considered the main mechanism by which PSB dissolves inorganic phosphate (Zuluaga et al., 2023). Yahya et al. (2021) showed that *Pseudomonas aeruginosa* could enhance effective soil phosphorus. He et al. (2022) found that phosphorus solubilizing bacteria can also convert phosphorus fixed in the soil into soluble forms through metal ion chelation and the presence of excess phosphate fertilizer. In this study, the phosphorus solubilizing capacity of NHP4a-2 and NHP4b-2 was evaluated. It was found that strain NHP4a-2 solubilized phosphorus up to 92.47 mg L^−1^ at pH 4.1 by day 7, while strain NHP4b-2 solubilized phosphorus up to 310 mg L^−1^ at pH 4.2 by day 3 (Figure 3). The phosphorus-solubilizing capacity of strain NHP4b-2 was greater than that of strain NHP4a-2. Therefore, we hypothesized that both strains might also be producing acids to facilitate phosphorus solubilization, aligning with previous studies by da Silva et al. (2021).

Biological phosphorus removal, which involves extracting phosphorus from phosphorus-rich wastewater using the metabolic mechanisms of phosphorus removal and recycling by PAOs (Zou and Wang, 2016). Saia et al. (2021) discovered that under anaerobic conditions, PAOs gain energy by hydrolyzing intracellular poly-P and releasing orthophosphate outside the cells. Liu et al. (2022) found that the phosphorus removal rate of PAOs incubated for 65 days reached 95%. Li et al. (2021) observed that under aerobic conditions, poly-P accumulated with bacterial growth, resulting in a decrease in the phosphorus concentration in the environment after 40 h of incubation. In our study, we found that when strains NHP4a-2 and NHP4b-2 were aerobically cultured at various concentration gradients, the phosphorus content in the medium gradually decreased. Also, the phosphorus content in the bacteria gradually increased with the growth of the bacteria (Figure 4). In addition, the higher the initial phosphorus concentration in the medium, the more phosphorus was absorbed by the bacteria as the incubation period extended (Figure 4). Notably, strain NHP4b-2 exhibited a superior ability to aggregate phosphorus compared to strain NHP4a-2. PAOs release phosphorus under anaerobic conditions and absorb it under aerobic conditions (Zhang et al., 2024). In this study, we found that both bacterial strains were more capable of releasing phosphorus under anaerobic conditions when high-phosphorus organisms were transferred to phosphorus-free medium. Under aerobic conditions, both bacterial strains absorbed phosphorus when cultured in low-phosphorus medium and then transferred to high-phosphorus medium (Figure 5). This further confirmed that the strains possess the characteristics of aerobic phosphorus absorption and anaerobic phosphorus release.

In bacteria, PPK expression level directly impacts the polyP capacity of PAOs (Rao et al., 2009), polyP degradation into Pi is primarily mediated by PPX (Akiyama et al., 1993; Bolesch and Keasling, 2000), which is crucial for Pi utilization and cycling in bacterial cells (Li and Dittrich, 2019; Hiyoshi et al., 2021). In this study, we found that PPK activity of strains NHP4a-2 and NHP4b-2 initially increased and then decreased when cultured aerobically in medium with varying phosphorus concentrations (Figure 6). The PPK activity decreased as the phosphate concentration in the medium decreased, with the highest activity observed at a phosphorus concentration of 4 g L^−1^, followed by 0.5 g L^−1^ and 0.1 g L^−1^. When the cultures were transferred to anaerobic incubation in phosphorus-free medium, PPX activity was measured, and it increased over time (Figure 6). Therefore, we hypothesized that PPX catalyzed the release of intracellularly stored poly-P into phosphate *in vitro* as the bacteria grew. Bacteria produce organic acids during phosphorus solubilization, with gluconic acid being the most common of them. In bacteria, GDH produces gluconic acid by directly oxidizing glucose (Suleman et al., 2018). Strain NHP4a-2 exhibited the highest GDH enzyme activity on day 7, while strain NHP4b-2 reached its highest GDH enzyme activity on day 3, followed by a gradual decrease and stabilization. The GDH activity of both bacterial strains aligned with the corresponding phospholysis trends.

The utilization of any phosphorus compound is divided into two processes: phosphorus uptake and the synthesis of ATP from inorganic phosphate (Oehmen et al., 2005a). Oxidative phosphorylation is the main pathway for ATP production (Oehmen et al., 2005b). The phosphate transport system’s substrate-binding proteins, ATP-binding proteins, and permease proteins have all been implicated in the transport of poly-P (Campos et al., 2019). To further explain the differences in gene expression between strains in the two states of phosphorus solubilization and phosphorus aggregation, this experiment was conducted by transcriptome analysis of the J4G and JCaG treatment groups. According to GO analysis, the genes were mainly involved in the “oxidation–reduction process,” “cellular component,” “transmembrane transportation,” and “structural constituent of the ribosome” (Figure 7D, Supplementary Table S2). The TCA cycle produces various organic acids (Akram, 2014; Vuoristo et al., 2016). Pyruvate was the main organic acid secreted during P solubilization (Zeng et al., 2017). The acidolysis pathway is considered a major mechanism in phosphate solubilization, with many organic acids involved in the microbial solubilization of insoluble inorganic P (Sashidhar and Podile, 2010; Ludueña et al., 2018; Li H. P. et al., 2023; Li Z. et al., 2023) The KEGG enrichment results of this study identified glycolysis/gluconeogenesis, oxidative phosphorylation, carbon metabolism, ribosome, pyruvate metabolism, flagellar assembly, and the citrate cycle (TCA cycle) as key pathways (Figure 7E, Supplementary Table S3). Four genes in the citric acid cycle (TCA cycle) pathway, which is closely related to organic acid production, were upregulated in JCaG (Supplementary Table S3), as well as nine genes in the pyruvate metabolism pathway. These genes play a significant role in inducing organic acid secretion by the strain. GO and KEGG results also showed that the strain secretes various types of organic acids to solubilize P. Based on GO and KEGG results, we hypothesized that glycolysis, the TCA cycle, and pyruvate metabolism produce ATP and acids that promote phospholysis in the strain. Under anaerobic conditions, the strain can hydrolyze ATP *in vivo* for energy while breaking down intracellular polyphosphate (Ploy-P), which is then released into the extracellular environment. The strain facilitates a slow release of phosphorus, promoting plant uptake by regulating different metabolic pathways that convert various forms of phosphorus in the body. Simultaneously, the transcriptome data reveal numerous candidate genes, such as high-affinity phosphate transporter genes and polyphosphate kinase genes. Investigating the functions of these genes provides conclusive causal evidence for explaining the phosphate solubilization or accumulation capabilities of the strain.

Rice, the world’s second largest food crop, faces challenges in absorbing excess phosphorus from the soil during its growth and development. The excess phosphorus from fertilizers cannot be fully utilized, leading to soil phosphorus eutrophication. Gupta et al. (2022) found that phosphorus-solubilizing bacteria (PSB) can mobilize phosphorus in the soil. Wang et al. (2022) further discovered that inoculating soil with PSB releases fixed phosphorus, forming phosphate that enhances phosphorus fertilizer utilization and promotes wheat growth. In this study, the application of both strains, NHP4a-2 and NHP4b-2, significantly increased the aboveground and belowground biomass of rice seedlings (Figure 9). This indicates that both strains improved phosphorus uptake and utilization in rice seedlings, with strain NHP4b-2 demonstrating a stronger phosphorus-solubilizing capacity than NHP4a-2. Under anaerobic conditions, phosphorus-accumulating organisms (PAOs) release large amounts of phosphorus, thus promoting plant growth (Tomás-Martínez et al., 2023). By inoculating strains NHP4a-2 and NHP4b-2 into culture media with different phosphorus concentrations, plants in the NHP4b-2 group exhibited higher phosphorus content, while phosphorus levels in the nutrient solution were lower than in the NHP4a-2 group (Figures 9B,C). We hypothesize that phosphorus released by the NHP4b-2 strain is more readily absorbed by plants, a phenomenon potentially linked to differences in the strain’s ability to produce plant growth-promoting substances such as indole-3-acetic acid (IAA). Extensive research confirms that IAA produced by plant growth-promoting rhizobacteria (PGPR) is vital for plant survival (Song et al., 2021; Aslam et al., 2022; Benmrid et al., 2014). Therefore, further validation of IAA production in both strains and observation of their actual effects on plant roots are warranted.

## 5 Conclusion

In this study, two phosphate-solubilizing and phosphate-accumulating bacterium, Acinetobacter johnsonii (NHP4a-2) and Pseudomonas glycinae (NHP4b-2) were isolated. These bacteria have the ability for aerobic phosphorus uptake and anaerobic phosphorus release. The maximum amount of dissolved phosphorus reached 92.47 mg L^−1^ for strain NHP4a-2 and 310 mg L^−1^ for strain NHP4b-2. The incubation of the strains at seven different phosphorus concentrations indicated that, as the initial phosphorus concentration in the medium and the number of incubation days increased, phosphorus concentration in the bacterium increased while those in the supernatant decreased. This indicates a gradual increase in phosphorus aggregation ability. Strain NHP4b-2 demonstrated stronger phosphorus solubilization and aggregation abilities than strain NHP4a-2. In addition, the PPK, PPX, and GDH activities of strain NHP4b-2 were higher than those of strain NHP4a-2. Transcriptome analyses show differential gene expression in strains during phosphorus solubilization and polymerization of phosphorus. In this study, the aboveground and belowground biomass of rice seedlings significantly increased with the application of NHP4a-2 and NHP4b-2 strains, indicating that both strains enhanced phosphorus absorption and utilization in rice seedlings. We believe that this study will provide insights for achieving efficient phosphorus cycling, addressing phosphorus resource scarcity and phosphorus pollution issues. It holds significant importance for reducing chemical phosphorus fertilizer application and developing slow-release bio-fertilizers, while also demonstrating the feasibility of utilizing the strains in agricultural production.

## Data Availability

All raw sequencing data have been uploaded to public databases to ensure reproducibility. Data are stored in the [NCBI Sequence Read Archive] database: https://www.ncbi.nlm.nih.gov/sra/PRJNA1173258.
