# Supplementary figures and images for: Phosphate-solubilizing and polymerizing bacteria enhance phosphorus availability and growth of rice
Source: Front Microbiol. 2025 Dec 8;16:1700135. doi: 10.3389/fmicb.2025.1700135 (PMC12719419; doi:10.3389/fmicb.2025.1700135)

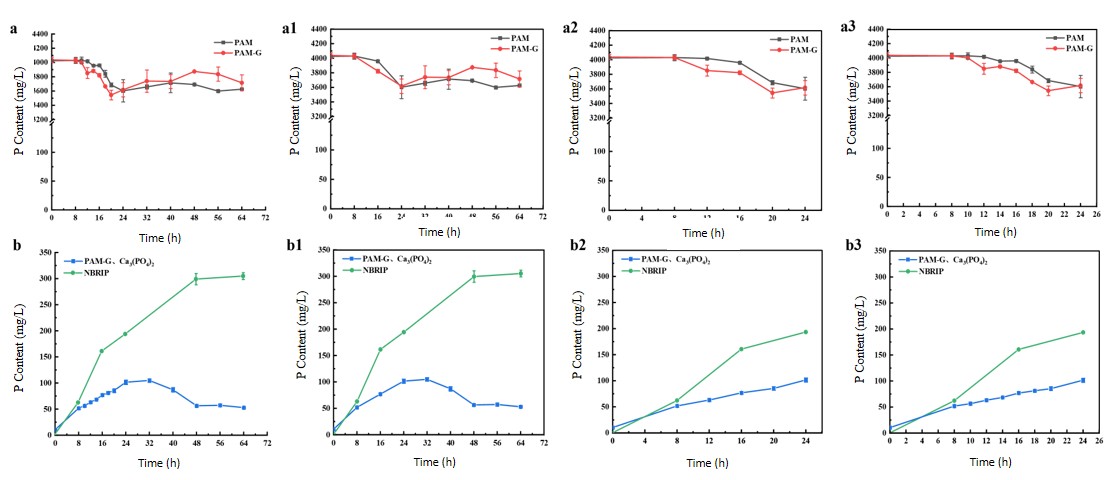

Supplement: Supplementary file 1 [file Image_1.JPEG]

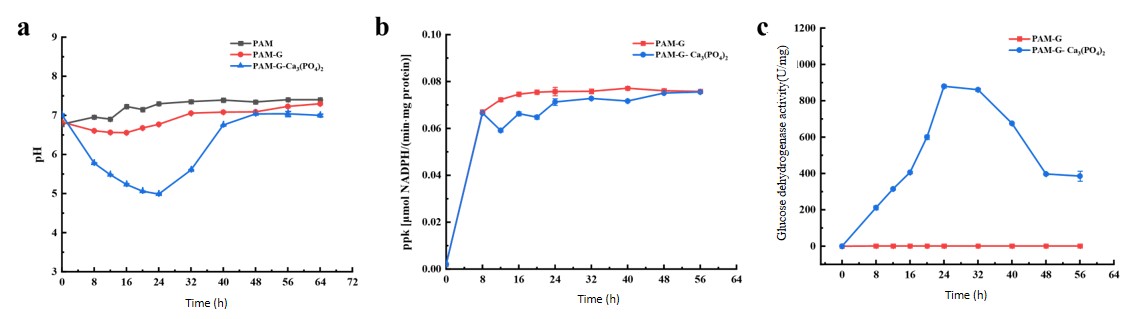

Supplement: Supplementary file 2 [file Image_2.JPEG]

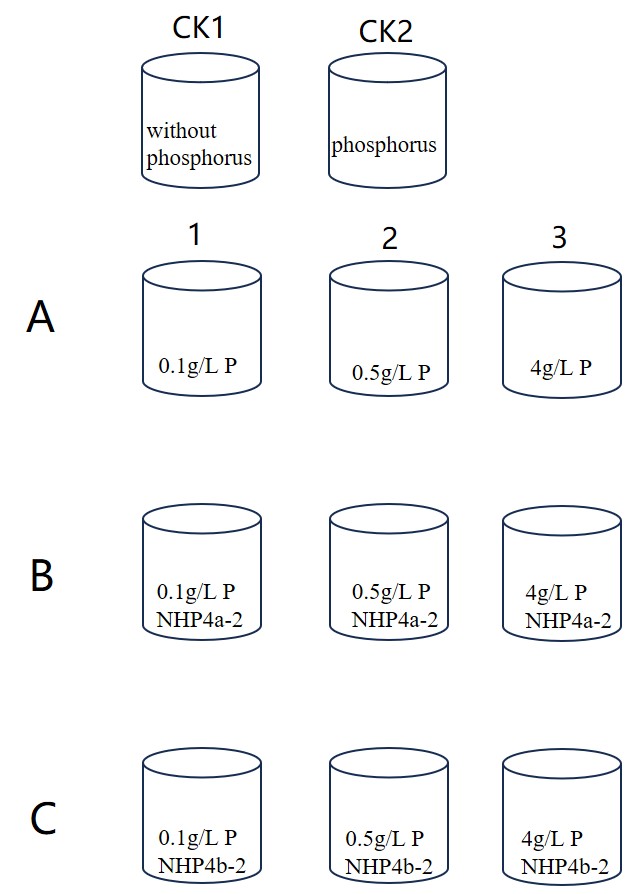

Supplement: Supplementary file 3 [file Image_3.JPEG]

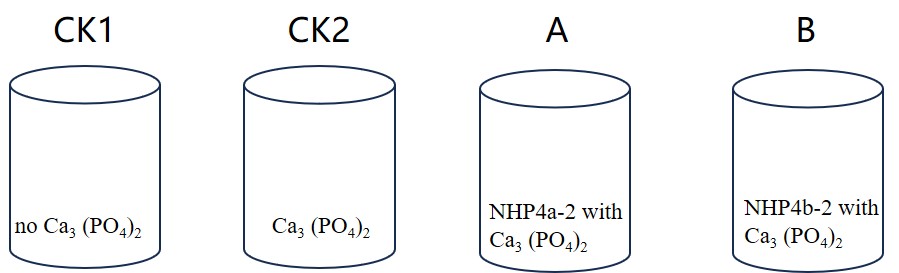

Supplement: Supplementary file 4 [file Image_4.JPEG]
