# Supplementary material for: Phosphate-solubilizing and polymerizing bacteria enhance phosphorus availability and growth of rice
Source: Front Microbiol. 2025 Dec 8;16:1700135. doi: 10.3389/fmicb.2025.1700135 (PMC12719419; doi:10.3389/fmicb.2025.1700135)
Supplement: Supplementary file 7 [file Table_3.DOCX]

Tab. S4 qPCR primer sequences

| 引物 | F/R（5’- 3’） |
| --- | --- |
| 16S rDNA | GGACGGGTGAGTAACGCGTA/CCCATTGCGGAAAATTCCCC |
| *ELZ14_RS09320* | GAGAAGACTTAAGCGTGATC/ TCAGCCGACGGCGTGAAGTT |
| *rpoH* | TGGAAGCCTACGTGCACTCG/ TTCATCAGGCCGACGTTGC |
| *secY* | TGAGTATCTTTGCACTGGGGA/TGTACTGGCTGATCTTGCG |
| *ELZ14_RS23605* | TCGATGGCGACCTGATCCAG/ GAGTAGTTCGGCTCGCGTTC |
| *atpA* | GCCCGTAACGAAGGCACTGT/ CCGCCCGGAAACTCGATCAT |
| *ELZ14_RS00830* | ATGGAACGTACACTCAGTTC/ TTAGCGCCAGAACGGCTTGC |
| *Flha* | GCGATGATGACCCTGCCGAT/ GCGTACACGCAGACCAGCAG |
| *atpD* | CAAGGTTATCGACCTGGTTT/ ATAACCGCTGTGCTCGATGG |
| *zwf* | ATGTCGGCTTGCGCGAGGCG/ GGTTCTCGCAGATGCCGCCG |
| *flgB* | GAGGTGTTGTCCAGCAACAT/ CAAGGCCAAAGCCCTGTTGT |
