# Supplementary material for: Phosphate-solubilizing and polymerizing bacteria enhance phosphorus availability and growth of rice
Source: Front Microbiol. 2025 Dec 8;16:1700135. doi: 10.3389/fmicb.2025.1700135 (PMC12719419; doi:10.3389/fmicb.2025.1700135)
Supplement: Supplementary file 8 [file Table_4.DOCX]

|  | **CK1** | **CK2** | **A** | **B** |
| --- | --- | --- | --- | --- |
| Plant height(cm) | 27.75±0.35d | 32.75±0.35c | 35.5±0.71b | 37.75±0.35a |
| Root length(cm) | 6.75±0.35d | 7.75±0.35c | 9.7±0.28b | 12.25±0.35a |
| Leaf fresh weight (g) | 1.29±0.01d | 1.38±0.03c | 1.66±0.04b | 1.88±0.04a |
| Root fresh weight (g) | 0.23±0.03d | 0.42±0.02c | 0.67±0.01b | 0.84±0.02a |
| Leaf dry weight (g) | 0.13±0.01c | 0.16±0.01b | 0.21±0.01a | 0.23±0.01a |
| Root dry weight (g) | 0.06±0.01c | 0.08±0.01bc | 0.1±0.01b | 0.13±0.01a |
| Length(cm) | 216.69±4.95d | 310.37±14.85c | 390.64±0.71b | 516.59±5.66a |
| Average diameter (cm) | 0.87±0.06b | 1.21±0.28ab | 1.21±0.01ab | 1.28±0a |
| Volume(cm3) | 9.89±0.28d | 15.05±0.71c | 19.93±0.71b | 32.63±2.83a |
| Surface area(cm2) | 119.38±10.61c | 161.99±12.02b | 199.09±1.41a | 212.92±4.24a |
| Projection area(cm2) | 37.61±2.83c | 50.86±2.83b | 64.69±1.41a | 67.82±1.41a |
| Number of root tips | 241±18.38c | 548.5±48.79b | 640.5±38.89b | 853±19.8a |

**Tab. S5 Effect of different treatments on the growth of rice seedlings**

**Different letters indicate significant differences p<0.05**
